# A Vibration-Based Methodology to Monitor Road Surface: A Process to Overcome the Speed Effect

**DOI:** 10.3390/s24030925

**Published:** 2024-01-31

**Authors:** Monica Meocci

**Affiliations:** Dipartimento di Ingegneria Civile e Ambientale, Università degli Studi di Firenze, 50139 Firenze, Italy; monica.meocci@unifi.it

**Keywords:** road pavement monitoring, vibration-based methodology, speed effect

## Abstract

Road pavement monitoring represents the starting point for the pavement maintenance process. To quickly fix a damaged road, relevant authorities need a high-efficiency methodology that allows them to obtain data describing the current conditions of a road network. In urban areas, large-scale monitoring campaigns may be more expensive and not fast enough to describe how pavement degradation has evolved over time. Furthermore, at low speeds, many technologies are inadequate for monitoring the streets. In such a context, employing black-box-equipped vehicles to perform a routine inspection could be an excellent starting point. However, the vibration-based methodologies used to detect road anomalies are strongly affected by the speed of the monitoring vehicles. This study uses a statistical method to analyze the effects of speed on road pavement conditions at different severity levels, through data recorded by taxi vehicles. Likewise, the study introduces a process to overcome the speed effect in the measurements. The process relies on a machine learning approach to define the decision boundaries to predict the severity level of the road surface condition based on two recorded parameters only: speed and pavement deterioration index. The methodology has succeeded in predicting the correct damage severity level in more than 80% of the dataset, through a user-friendly real-time method.

## 1. Introduction

Road networks extend for thousands and thousands of kilometers and very often provide a snapshot of the income level of the country where they are located. Distressed and/or perfect pavement surfaces provide immediate insight into the budget invested in road maintenance by Road Agencies (RAs). The progressive increase in road pavement distress is a natural phenomenon, and as people age, so do the roads. As time passes, however, they must be promptly “treated” to slow down the aging process and maintain an acceptable and safe condition for all road users. Low friction levels, roughness, permanent deformations, and potholes are different types of distresses generally visible on the pavement surface. The more the level of distress severity increases, the more these phenomena are visible and could affect driving comfort and traffic safety.

As mentioned, age is one of the most important variables affecting road distress evolution in terms of severity and decay speed. The cost of maintenance intervention and pavement restoration accordingly increase.

Therefore, an RA needs low-cost management plans to detect the most distressed sites and quickly arrange appropriate interventions that mitigate and repair the road pavement anomalies before they become dangerous for traffic.

The common Pavement Management System (PSM) is based on a monitoring system capable of describing the road surface quality. This process is normally conducted by inspections, which, however, represent a time-consuming and subjective approach that directly exposes administration personnel to the risk of traffic. Sometimes, the monitoring phase is conducted by high-performance devices that allow the RA to survey the entire road pavement structure, both in terms of ride comfort and structural performance. The extensive use of this methodology, very accurate and based on a combination of image and sensor data processing, can also be very expensive. Therefore, an alternative low-cost monitoring method needs to be identified, certified, and used, especially for routine monitoring.

Lekshmipathy et al. (2020) [1] define the vibration-based methods as the most effective in the daily monitoring of road network conditions. If appropriately integrated with both more accurate monitoring methods such as the vision-based methods for local monitoring and distress trends, the mentioned methodologies offer the best solution in terms of repair and maintenance interventions to be performed in accordance with the detected pavement surface distress level.

The Global Pave Box (GPB) technique proposed by Meocci et al. (2021) [2], which has been tested on several road sections within the Florence Municipality [3,4], could represent a good solution in the daily collection of pavement surface data. However, the vibration-based method, as defined in scientific literature, is strongly dependent on the speed of the equipped vehicles [5,6]. Consequently, the data recorded are affected by bias and can sometimes not be used due to the difference in vehicle speed during the monitoring process.

In this context, the need to both perfect the methodology proposed in 2021 by Meocci et al. [1] and make it reliable and immediately applicable at the same time has arisen. The possibility of using a crowdsourcing approach directly applicable to the road network has therefore become the main objective of this study. In these terms, both the definition of the influence in the proposed methodology of the monitoring speed and the effect of speed values on the descriptive indicators of the road surface condition are investigated. At the same time, the research proposes a simple methodology to overcome the issue, making a road section immediately classifiable by only two parameters: monitoring speed and the distress index (GPB index).

The analysis carried out also defines the limitations of the process proposed.

## 2. State of the Art

Starting in the last decade, many studies have been conducted on the possibility of assessing road pavement conditions by employing a sensor (such as a black box, accelerometer, or smartphone) to be used inside cars.

The premise is that when a vehicle circulates on a distressed road surface (e.g., characterized by potholes, cracking, and unmaintained manholes), it produces more vibrations compared to a perfectly maintained road pavement surface [5]. This is the principle used both for the evaluation of standardized index evaluation, such as the IRI index [7,8,9,10], and also with reference to non-standardized procedures and indices [11,12,13,14,15,16].

In 2008, Eriksson et al. investigated the possibility of detecting road surface distress using the Pothole Patrol (P2) system, an application that uses taxis equipped with mobile sensors (accelerometers and GPS sensors) and the associated algorithms to define road surface conditions according to vibration data [17].

Similar techniques named Road Condition Monitoring with Three-axis Accelerometers and GPS Sensors (RCM-TAGPS) were used and developed by Chen et al. in 2011. In this case, a three-axis accelerometer and a GPS-sensor-equipped vehicle were used to collect high-frequency data. The data recorded were analyzed by a Power Spectral Density to evaluate the roughness of the road [18].

In the same years, similar studies were conducted using the accelerometer and the sensor inside the smartphone. For example, Mohan et al. (2008) proposed the Nericell system, an application that records traffic and road conditions using data recorded by the sensors on smartphones that users carry with them when they drive a car [19]. Other studies investigated the road surface status with GPS-equipped smartphones and inertial sensors: accelerometer and gyroscope or other specific applications developed to monitor the road with the smartphone [20,21,22,23,24].

In Sri Lanka, they developed a crowdsourcing method, called Bus Net, capable of improving environmental surveying and providing information about road pavement conditions [25].

In recent years, this topic has been extensively debated, with the consideration of new data analysis techniques such as Machine Learning and Artificial Intelligence [12,26,27,28,29]. Martinelli et al. processed the real data collected by sensors located on car dashboards and floorboards, employing decoding algorithms machines. These propose a plausible technology capable of detecting any kind of road surface distress and ranking it according to a data clusterization based on the coefficient of variation and entropy [12].

Shtayat et al. used two multiclassification Learning Model Machines (Random Forest and Decision Tree) to classify and automatically detect different kinds of pavement distresses. Moreover, their research with a Support Vector Machine algorithm developed a binary model for the same classification and detection purposes [26].

Other authors instead compared different methods to define the pros and cons. For example, Kim et al. analyzed pothole detection with vibration, vision, and 3D reconstruction methods [30]. Dib et al. instead compared deep learning techniques and non-deep learning techniques for detecting damaged road surfaces [31].

Ferjani instead stated that there are several systems based on the data collected by sensors inside the cars, especially with reference to accelerometers present in smartphones, but these methods require great attention in the dataset elaboration to provide accurate and reliable results that also include, for example, information regarding time-, frequency-, and wavelet-domain signal features. In his study, the problem was addressed using machine learning models to detect and classify the road anomalies [27].

As described in the review conducted by Coenen et al. in 2017 [32], a delay in the diffusion of the vibration-based methods was recorded due to complex data analysis affected by multiple variables. It was accordingly impossible to offer the market a reliable approach [27] when compared with a vision-based technique that is more expensive but more popular in the Road Surveying Market.

Sensor features, such as vehicle model, kind of indicator used to check a road surface condition, and survey speed, are only a few of the main variables affecting the vibration process. In particular, the latter is more effective in the case of a crowdsourcing approach used for data collection.

Acceleration values and the different speed performed in different road contexts, but also in the same context, for example, the urban road network can be very different.

In past studies, the speed effect on the quality of the vibration-based methodology was both analyzed and sometimes eliminated by normalization procedures. When a vehicle runs over a road distress area, e.g., such as a pothole or an unmaintained manhole, at different speeds, the amplitude of the vertical acceleration collected can change, first in amplitude but also in frequency due to the different effects of the vibration induced by road surface on the vehicle itself. A few approaches to solve the problem propose modelling of that effect; for example, using a theoretical model including road profile, tires, and suspension. In the study conducted by Alessandroni et al., it was demonstrated that the speed effect on the acceleration in the vehicle cabin was identified by a gamma function [33].

Fox et al. evaluated the direct effects of speed on road distress detection, proving that high speed increases the level of difficulty in ranking road pavement conditions [34].

Many approaches consider speed as included in different ranges and not as a continuous function. Sebestyen et al. and Sinharay et al., for example, normalized the result obtained according to speed ranges [35,36]. The same approach was proposed in [9], where the authors normalized the RMS of the signal by the variance of the signal itself (vertical acceleration), as proposed in other studies conducted by Zeng et al. [37] and Chou et al. [38].

Seraj et al., on the other hand, processed smartphone data according to time domain, frequency domain, and wavelet transformation. They also proposed an efficient methodology to reduce speed effects’ influence in detection and classification of road pavement distress, by amplifying the segment of the signal caused by the distress in accordance with vehicle speed [39].

In any case, the most popular approach proposed different algorithms for different speeds [19,24,39,40,41], effectively making the proposed systems very difficult to apply in the different road context because the difference in variable and procedure are strongly dependent on the road type (e.g., rural or urban), either in reference to standard indicators (e.g., IRI) or by means of specific indicators [42,43,44]. The same results appear in reference to the classification of distress carried out using a machine learning approach.

At first, the research to which this article refers was conducted forcing the vehicles’ speed in the range defined by the literature review as the most frequent speed in urban areas, e.g., which range from 30 km/h to 40 km/h [2,3,4]. All the same, considering that the literature analysis offers several different techniques too different from one another and not robust enough to account for the effect of a vehicle’s speed, in this paper, the effect of the speed was first analyzed in terms of statistical process. After this, a simple process based on machine learning algorithms was used to determine decision boundaries for different severity levels in a simple way and offer an inexpensive and user-friendly approach to analyzing road surface conditions and their influence on the speed effect.

The approach allows to extend the validity of the proposed methodology at least at all the different speeds performed in the urban context.

## 3. Methods

### 3.1. Experimentation Overview

**Figure 1:**
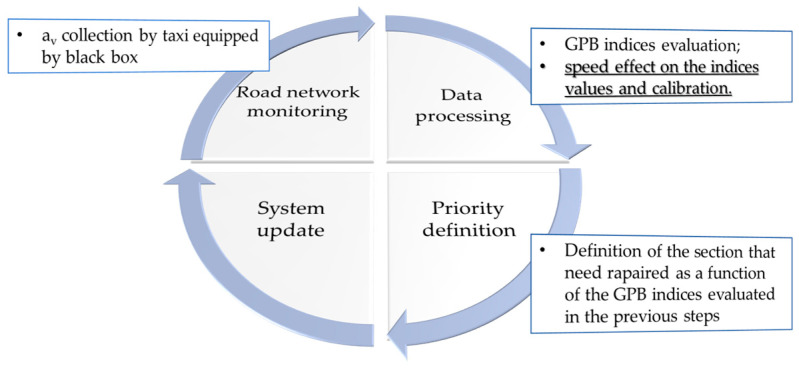
Monitoring steps.

The procedure defined for the road network screening is summarized in Figure 1. It consists of monitoring the road network (or road section as in this case) and recording the vertical acceleration on the taxis equipped with the black box that routinely pass across the road network.

Data processing and last classification of the road networks maintenance are completed by the procedure based on the distress index evaluation (GPB index). Each time one road segment was covered by a taxi passage, the monitoring process offers information about the pavement condition for this specific segment within the overall road network. Consequently, with this information, the segment information in the overall system needs to be updated.

The proposed system constitutes a starting point to manage and maintain roads pavement, especially for the urban context.

### 3.2. Monitoring Procedure and Evaluation Index

Ten Toyota Prius Hybrid 1.8 taxis equipped with black boxes were used for a period of 15 months to record vertical acceleration along four road segments within the Florence Municipality road network.

The black box used consists of one simple device composed of one inertial accelerometer able to record acceleration in all the three directions, X, Y, and Z, one triaxial gyroscope, and a GPS device. The device was characterized by a sampling rate of 0.01 s in time (100 Hz). This frequency is consistent with the sampling frequency of the devices used and described in past research [2,17]. For each direction, the maximum acceleration recordable is equal to 16 g, representing a very high value in comparison to the vibrations (and accelerations) recorded by the distressed road surface.

**Figure 2:**
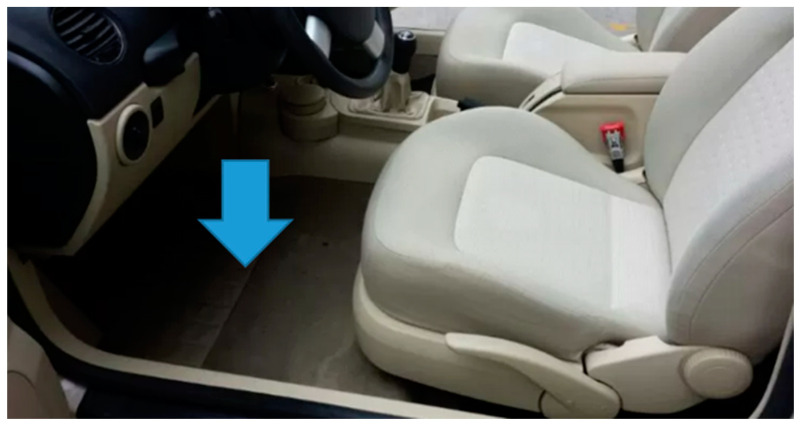
Black box position.

The black box is connected to the car floorboard, between the pedals and gearbox, as close as possible to the car center of gravity in order to minimize centrifugal/centripetal force effects on the recorded signal, as shown in Figure 2.

This device does not require any installation and calibration procedure. As GPS connection is stabilized, the taxi can regularly travel according to clients’ requested destinations.

Road sections were tested at speeds ranging between 20 km/h and 50 km/h.

**Figure 3:**
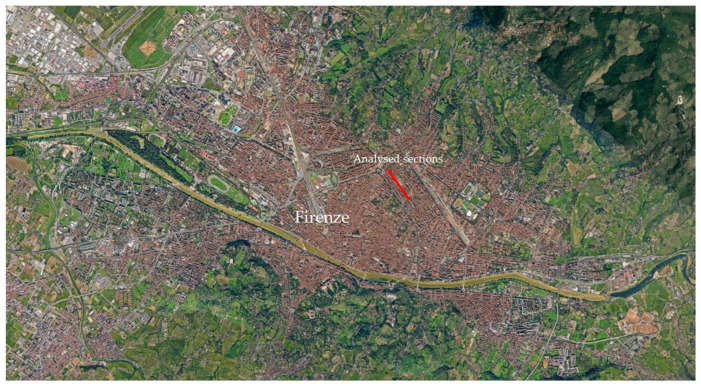
Road sections assessed.

Sometimes, the speed recorded was a bit less or greater than this range, but consistent with the driver’s speed within the urban context. In Figure 3, the road sections analyzed are shown (in red).

The data recorded by the monitoring were used both for the identification and classification of the pavement surface damage and the definition of damage severity by an iterative procedure.

The procedure proposed was based on the evaluation of the vertical acceleration value recorded during the road monitoring (when taxis run across the road network). A simple algorithm allows the estimation of the Global Pave Box Index (GPB), as described in Meocci et al. [2]. The GPB index evaluation is repeated every time data are provided for the considered road section and updated by calculating the average value considering the “i” passages conducted on the same section. The larger the number of taxi passages, the greater the reliability and correctness of the procedure.

The GPB index is based on Equation (1).

(1) $$GPB=\left(\int_{0}^{L}{\hat{\sigma \left(l\right)}}^{2}dl\right)\times \frac{100}{L}$$

where ${\hat{\sigma \left(l\right)}}^{2}$ stands for the moving variance of vertical recorded acceleration (a_v_), and L represents the total length of road segment. The term 100/L allows to compare road sections characterized by different length as a function of the standard length of 100 m long.

Equation (2) describes the moving variance estimation.

(2) $$\hat{\sigma^{2}}=\frac{\sum_{i=1}^{n}{\left(a_{v,i}-\bar{a_{v}}\right)}^{2}}{n}$$

where a_v,i_ is the vertical acceleration recorded every 0.01 s, and $\bar{a_{v}}$ is the average value estimated for a number of n = 20 a_v_ values. This process allows to eliminate the noise of the signal by a filtering process using the moving variance over a sliding window of 0.2 s length (20 a_v_ data), across the central range value. The process allows to align the precision of the information contained in the acceleration signal according to the GPS accuracy in the distress location (10 m).

As a function of the GPB values obtained, three severity levels were defined to describe the pavement condition: low, medium, and high (Table 1).

These levels characterize the condition of the road pavement to be considered in the definition of a priority plan for maintenance and restoration operations on the entire road network.

A fourth and final level must, however, be defined for all those cases in which the intervention has to be carried out immediately for road safety. In this case, the index does not contribute to the prioritization of the intervention but gives to the RAs an alert defined as the immediate “need for repair”.

**Figure 4:**
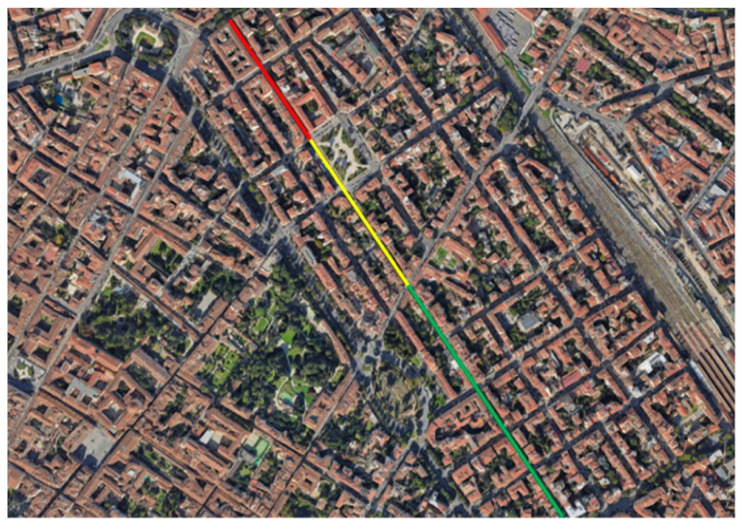
Road sections assessed.

The entire path monitored was divided into four sections characterized by different distress levels based on the GPB index, as shown in Figure 4. One road segment was included in the high severity level (red), one segment in the medium severity level (yellow), and the last two segments were classified as low severity (green).

### 3.3. Data Processing

The distress index evaluated (GPB) was used to assess the speed impacts on the evaluation process and understand how survey speed affects this proposed methodology, in accordance with literary research findings.

The analysis was conducted by dividing the dataset into three different classes, one for each severity level (low, medium, and high). This was conducted in accordance with the four segments monitored in the research, the pavement conditions of which belong to different distress severity classes (one segment was classified in high severity, one in medium severity, and the last two in low severity).

The speed effect was then analyzed by statistical approach, with the road surface distress, evaluated in terms of GPB index considered as a dependent variable.

A preliminary Levene’s test was performed to assess the equality of variances (homoscedasticity) of the dataset used for the statistical analysis. Then, a one-way ANOVA test was performed to evaluate whether there were statistically significant effects of the speed on the dependent variable. First, the ANOVA test was performed on the whole dataset, and later repeated three times, one for each road segment sample, considering implicitly the segment severity distress level and next combining the 2 classified segments in the same level (low severity).

Speed was ranked in three different ranges <30 km/h, 30–40 km/h, and 40–50 km/h, respectively named 1, 2, and 3 in the analysis. The independent variable was then represented by the speed range (1, 2, or 3) associated with each GPB index obtained.

The two following hypotheses were postulated and evaluated by the ANOVA test:

(a) null hypothesis (H_0_): GPB values obtained from the three speed ranges can be considered as belonging to the same population, hence speed surveying does not affect the results in terms of severity classification;
(b) alternative hypothesis (H_1_): the GPB index values do not belong to the same population; therefore, there is a significant difference between the GPB value estimated at different speeds. In this case, the classification of the road pavement condition needs to be made carefully, with reference (only) to a specific range of survey speed.

In the statistical analysis, a level of significance equal to 95% was selected.

Because the scientific and technical literature highlight a large dependence of the vibration-based methods on the speed of the vehicle which conducted the monitoring [5] and also highlight the issues related to different processes to consider the effect of the variable in both the distress detection and classification, a simple, replicable, and reliable procedure was proposed to take into account the speed effect.

A cluster analysis to classify the information obtained by the data processing procedure was finally proposed. In this last step, the entire dataset was divided as a function of the severity level and the decision boundaries between the severity levels defined by a Support Vector Machine (SVM) algorithm to directly obtain the classification of the road surface distress by the couple represented by speed-GPB values without any correction.

## 4. Results and Discussion

For each section, the value of the GPB index was evaluated according to the above briefly described procedure proposed by Meocci et al. [2]. A total of 926 GPB indices was considered. Table 2 shows the average value of the GBP index for each section and the descriptive statistics associated with the considered section.

**Figure 5:**
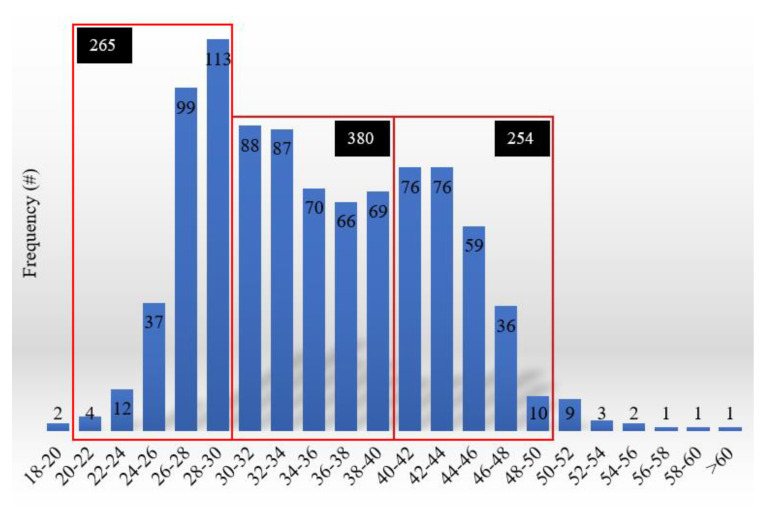
Speed distribution.

According to the dataset, the speed range between 30 km/h and 40 km/h was the most representative for the survey conducted in an urban area. Figure 5 shows speed distribution.

**Figure 6:**
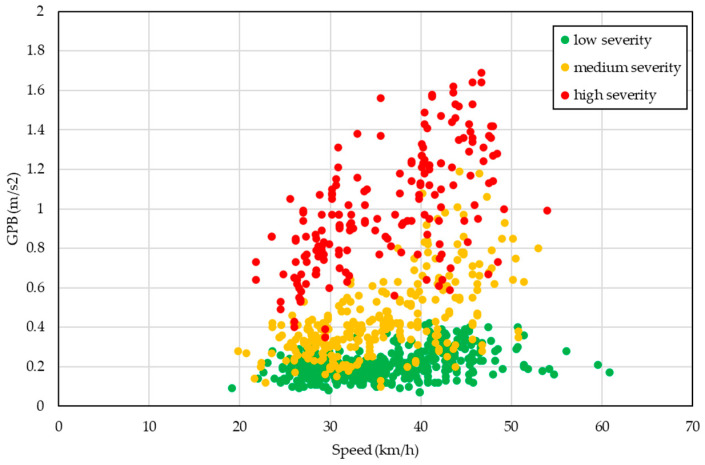
GPB index—speed per segments.

In Figure 6, all the GPB evaluated were plotted as a function of the speed. The colors used referred to the severity of the pavement distress per segment. Segments 3 and 4 were considered together (green data).

From a quality point of view, Figure 6 shows a data stratification, especially in low/high distress levels. The phenomenon was instead not particularly evident for GPB included in the medium severity class, with scattered data that also overlapped with the other severity classes.

The ANOVA test conducted was based on two assumptions:

- Normally distributed data (Shapiro–Wilk test);
- Homoscedasticity of the data (Levene’s test).

The two tests were conducted on the dataset, first considering all data, then per each severity class. In both tests, the estimated *p*-value was less than 0.05; therefore, both hypotheses were violated.

The ANOVA test was usually robust, so the test was conducted, but in association with the Kruskal–Wallis nonparametric test.

Table 3 shows the result of the ANOVA test conducted. The *p*-value indicates that the H_0_ hypothesis cannot be rejected; therefore, somewhere in the three speed classes were data that did not show statistically significant differences.

The analysis was completed by a pairwise post hoc Tukey’s test, to check for any difference in the meaning of all possible pairs using a studentized range distribution. The results obtained are summarized in Table 4.

The test found a statistically significant difference between speed classes n. 1 and n. 3 and between the speed classes n. 2 and n. 3, as highlighted in bold in Table 4. Instead, no significant difference was found between speed classes 1 and 2. Therefore, the analysis conducted with a speed ranging from a minimum value of 20 km/h to 40 km/h did not affect the GPB results. Instead, the GPB value related to the high value of monitoring speed, greater than 40 km/h, affects the value of the index evaluated and therefore could change the severity estimations.

The nonparametric Kruskal–Wallis test confirms the result obtained in the ANOVA test. In Table 5, the results obtained are summarized.

This test was repeated after arranging the dataset per severity class, high, medium, and low, including data for segments 3 and 4. The two hypotheses were invalidated, so both ANOVA and Kruskal–Wallis tests were repeated. Table 6 summarizes the results obtained.

The tests allowed us to observe that, within each severity class, a statistically significant difference was observed for different classes of speed, except for the low severity distress class, where a statistically significant difference was not detected for speed ranging in classes 1 and 2. The results obtained are therefore similar to those obtained in the first statistical analysis.

The Support Vector Machine (SVM) analysis for the definition of the decision boundaries was repeated two times, the former considering linear decision boundaries and the latter considering power functions decision boundaries (Equations (3) and (4)). In each analysis, the overall dataset was split into a training set (80%) and a test set (20%).

Y = ax + b(3)

Y = ax^2^ + bx + c (4)

Two decision boundaries were proposed to cluster data in the different severity distress conditions. Ten iterations were repeated to split the dataset in different and casual way. The coefficients obtained for the functions in each iteration conducted are summarized in Table 7.

The ability of the model to correctly predict the cluster per each iteration is summarized in Table 8, both for linear and power functions decision boundaries.

**Figure 7:**
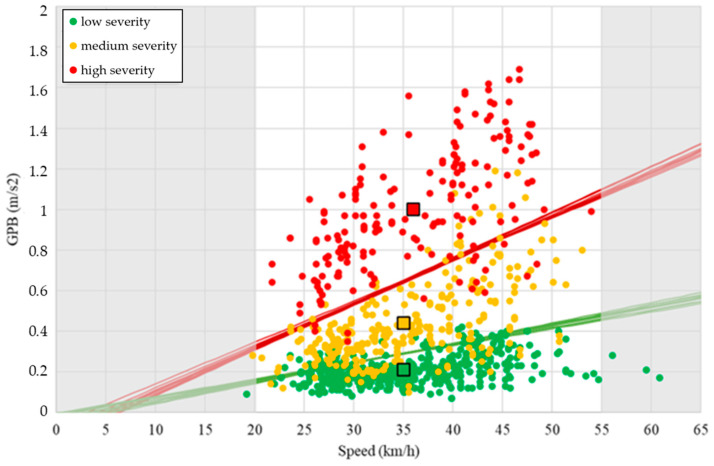
Result obtained with linear decision boundaries.

**Figure 8:**
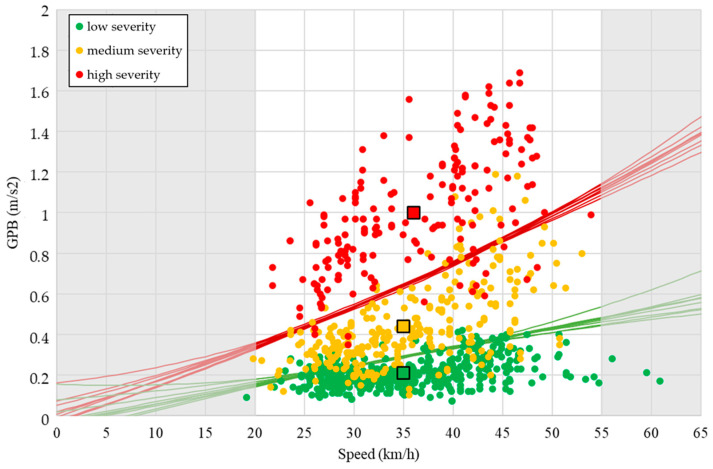
Result obtained with parabolic decision boundaries.

Table 8 shows a good ability to propose decision boundaries to predict the severity class of the road condition surface given the information about GPB and speed. The minimum value was observed for the medium severity level, which had an average value equal to about 77%. The values for the other classes were instead higher, and equal to 90% and 87%, respectively, for low and high severity. There were no differences between linear and parabolic functions to be highlighted. Figure 7 and Figure 8 show all the decision boundaries found. The accuracy obtained with this procedure is similar to, or greater than, those obtained in other literature research conducted by machine learning and artificial intelligence approaches [5,6,26,27].

The two graphs showed that both parabolic and linear decision boundaries allow a good prediction of the severity level of the data couple represented by speed and GPB. However, it is important to highlight that it is not appropriate to extrapolate the prediction outside the limits of the speed range described by the dataset. Indeed, for the lower limits, we can note that the linear function, for speed values close to 0, cannot be used due to the inversion of the two decision boundaries. On the contrary, the parabolic function tends to diverge a lot for high-speed values. Therefore, it is necessary to highlight that the procedure proposed needs to be limited to a relevant speed range between 20 km/h and 55 km/h which, in any case, covers the speed range usually performed (and also permitted) in the urban context.

## 5. Conclusions

Nowadays, many monitoring methodologies are available; some of them are characterized by high performance but also high costs, and others are characterized by limited and subjective performance and high time consumption. The vibration-based system called Pave Box consists of a user-friendly and cheap method based on the data crowdsourcing approach. Data collection uses the inertial devices (black boxes) located inside the taxi vehicles that routinely accompany passengers in the road context of the Florence Municipality. A simple algorithm allows the users to estimate the road surface distress based only on vertical accelerations recorded by the inertial devices.

Many other procedures similar to the Pave Box methodology have been proposed by scientific literature, but none are currently directly applicable and widespread enough in the area to carry out low-cost road pavement monitoring.

In this context, the need both to perfect the methodology proposed in 2021 and make it reliable and immediately applicable has allowed for improvements in the research dedicated to the analysis of the speed effect on the method results.

First, by means of a statistical analysis, the significance of speed in the distress index evaluation was estimated. Then, a simple and structured process to overcome the effect of speed in the severity level evaluation is proposed. The procedure is based on the data stratification for each severity level, only as a function of the monitoring speed. The use of a Support Vector Machine (SVM) algorithm allowed the definition of the decision boundaries between the different severity levels and the direct identification of the road segment pavement condition in terms of distress severity through only two data elements, speed and GPB index. The ability to correctly predict the severity level range as a function of the decision boundaries and the average values is close to 90%, except for the intermediate distress severity, where a 77% accuracy was defined.

These tangible results promoted the use of the process in the urban area.

However, the topic investigated in this paper leaves open several future developments, where the need to study the effect of different vehicles in the distress index evaluation undoubtedly represents the first challenge. Finally, the definition of a validation procedure that compares the results obtained with those obtained by the use of image-based methods, very precise and accurate, could complete and confirm what has been investigated to date.

## Figures and Tables

**Table 1:** Severity levels for GPB index.

GPB Value	Severity Level	
<0.40		low
0.40–0.65		medium
0.65–1.45		high

**Table 2:** Descriptive information on data recorded in monitoring.

Section ID	Sample Size	Average Speed (km/h)	Speed S.D.	Average GPB Index (m/s^2^)	GPB Standard Deviation
1	184	36	7.45	1.00	0.30
2	276	35	7.15	0.44	0.20
3	231	35	7.05	0.19	0.07
4	235	35	7.07	0.22	0.06

**Table 3:** Results of the ANOVA test.

Speed Class	F	*p*-Value
Between groups	14.762	**<0.001**

**Table 4:** Results of the Tukey post hoc test.

Speed Class		*p*-Value
1	2	0.995
	3	**<0.001**
2	1	0.995
	3	**<0.001**
3	1	**<0.001**
	2	**<0.001**

**Table 5:** Results of the Kruskal–Wallis test.

Speed Class	Statistica	*p*-Value
1–2	0.793	0.428
1–3	−5.541	**<0.001**
2–3	−4.437	**<0.001**

**Table 6:** Results of the Tukey post hoc test.

Level of Severity	Speed Class		*p*-Value ANOVA	*p*-Value Kruskal–Wallis
High	1	2	**<0.001**	**<0.001**
		3	**<0.001**	**<0.001**
	2	1	**<0.001**	-
		3	**<0.001**	**<0.001**
	3	1	**<0.001**	-
		2	**<0.001**	-
Medium	1	2	**0.007**	**<0.001**
		3	**<0.001**	**<0.001**
	2	1	**0.007**	-
		3	**<0.001**	**<0.001**
	3	1	**<0.001**	-
		2	**<0.001**	-
Low	1	2	0.722	0.452
		3	**<0.001**	**<0.001**
	2	1	0.722	-
		3	**<0.001**	**<0.001**
	3	1	**<0.001**	-
		2	**<0.001**	-

**Table 7:** Result of the iteration.

Boundary	Linear Function		Power Function		
	a	b	a	b	c
L-M	0.0086	−0.0126	−0.0001	0.0130	−0.0902
M-H	0.0219	−0.1298	0.0002	0.0051	0.1615
L-M	0.0100	−0.0574	0.0002	−0.0020	0.1549
M-H	0.0226	−0.1446	0.0001	0.0162	−0.0323
L-M	0.0092	−0.0294	−0.0001	0.0135	−0.0080
M-H	0.0204	−0.0624	0.00004	0.0173	−0.1058
L-M	0.0083	−0.0036	0.0001	0.0096	−0.0273
M-H	0.0214	−0.1083	0.00002	0.0110	0.0726
L-M	0.0096	−0.0477	0.000002	0.0098	−0.0503
M-H	0.0210	−0.0884	0.0001	0.0155	0.0071
L-M	0.0085	−0.0064	0.0001	0.0038	0.0770
M-H	0.0232	−0.1674	0.0003	−0.0012	0.2543
L-M	0.0089	−0.0182	0.00003	0.0069	0.0165
M-H	0.0225	−0.1343	0.0001	0.0137	0.0178
L-M	0.0086	−0.0109	0.00004	0.0117	−0.0667
M-H	0.0225	−0.1184	0.0001	0.0169	−0.0330
L-M	0.0095	−0.0397	0.000003	0.0092	−0.0354
M-H	0.0225	−0.1410	0.0001	0.0160	−0.0263
L-M	0.0091	−0.0273	0.00001	0.0101	−0.0445
M-H	0.0214	−0.1045	0.0001	0.0126	0.0490

**Table 8:** Ability to predict the cluster (severity level).

Iteration	Class	Linear		Power	
		Right	Non-Right	Right	Non-Right
1	L	85 (91%)	8 (9%)	85 (91%)	8 (9%)
	M	40 (82%)	9 (18%)	40 (82%)	9 (18%)
	H	37 (84%)	7 (16%)	37 (84%)	7 (16%)
2	L	79 (88%)	11 (12%)	80 (88%)	11 (12%)
	M	40 (70%)	17 (30%)	40 (71%)	16 (29%)
	H	35 (90%)	4 (10%)	35 (90%)	4 (10%)
3	L	80 (85%)	14 (15%)	80 (85%)	14 (15%)
	M	38 (69%)	17 (31%)	39 (70%)	17 (30%)
	H	34 (92%)	3 (8%)	34 (94%)	2 (6%)
4	L	83 (93%)	6 7%)	83 (93%)	6 (7%)
	M	42 (75%)	14 (25%)	43 (75%)	14 (25%)
	H	34 (83%)	7 (17%)	34 (85%)	6 (15%)
5	L	83 (90%)	9 (10%)	83 (90%)	9 (10%)
	M	40 (71%)	16 (29%)	40 (71%)	16 (29%)
	H	32 (84%)	6 (16%)	32 (84%)	6 (16%)
6	L	85 (89%)	10 (11%)	85 (89%)	10 (11%)
	M	39 (78%)	11 (22%)	40 (78%)	11 (22%)
	H	35 (85%)	6 (15%)	35 (88%)	5 (13%)
7	L	89 (90%)	10 (10%)	89 (90%)	10 (10%)
	M	41 (82%)	9 (18%)	41 (82%)	9 (18%)
	H	33 (89%)	4 (11%)	33 (89%)	4 (11%)
8	L	87 (91%)	9 (9%)	87 (91%)	9 (9%)
	M	39 (80%)	10 (20%)	39 (80%)	10 (20%)
	H	34 (83%)	7 (17%)	34 (83%)	7 (17%)
9	L	88 (94%)	6 (6%)	88 (94%)	6 (6%)
	M	46 (84%)	9 (16%)	47 (84%)	9 (16%)
	H	34 (92%)	3 (8%)	34 (94%)	2 (6%)
10	L	83 (88%)	11 (12%)	83 (88%)	11 (12%)
	M	41 (77%)	12 (23%)	41 (77%)	12 (23%)
	H	36 (92%)	3 (8%)	36 (92%)	3 (8%)

## Data Availability

Data are contained within the article.
